# Molecular assessment of accessory gene regulator (*agr*) quorum sensing system in biofilm forming *Staphylococcus aureus* and study of the effect of silver nanoparticles on *agr* system

**Published:** 2018-02

**Authors:** Ahmed Mohammed Turkey, Khadija Khalil Barzani, Ahmed Abdul Jabbar Suleiman, Jenan Jameel Abed

**Affiliations:** 1 Biology Department, College of Sciences, University of Anbar, Ramadi, Iraq; 2 Biology Department, Education College, Salahaddin University-Erbil, Erbil, Iraq

**Keywords:** *Staphylococcus aureus*, Biofilm, *Agaricus compestris*, AgNPs nanoparticles

## Abstract

**Background and Objectives:**

*Staphylococcus aureus* is an opportunistic human pathogen that causes a variety of diseases. Staphylococcal biofilms are a source of chronic and continual infections. This study was conducted to estimate the distribution of *agr* among different isolates of *S. aureus* and their relationship with biofilm. Also, it was aimed to check the association of operon *agr* with virulence factors (*seb, eta, spa* and *tst v8*) and study the effect of biosynthesis silver nanoparticles on the function of the *agr* system.

**Materials and Methods:**

Out of 580 clinical specimens, 100 *S. aureus* isolates were isolated and identified based on cultural, morphological, and different biochemical tests, in addition to molecular identification using PCR with specific primer *16SrRNA*. For biofilm detection, the fungi synthesized silver nanoparticles were used to check its effect on *agr* system.

**Results:**

The biofilm producer among *S. aureus* was 61% and non-biofilm producer isolates were 39%. It was found that the total number of *agr* - bearing isolates was 31 (50.82%), with a significant difference in the distribution percentage of virulence factors genes in isolates of biofilm-forming *S. aureus* carried *agr*. The results also revealed a relationship between the *agr*-quorum sensing system and the prevalence of virulence genes in the isolated *S. aureus*. Silver nanoparticles (AgNPs) were synthesized by *Agaricus compestris*, and it was found that it activates the *agr* system in 31 (100%) of biofilm-forming and carrying operon *agr* after treatment with sub-MIC of AgNPs.

**Conclusion:**

The findings of this study revealed that not all isolates of *S. aureus* have *agr* system. Also, it was found that AgNPs have a positive effect on bacterial virulence factors production and could be used for treatment or in cooperation with antibiotics to decrease resistance

## INTRODUCTION

Biofilms are sets of microorganisms in which cells affix to each other on a surface that is a polymeric mixture generally composed of proteins, extracellular DNA and polysaccharides (1). Staphylococcal biofilms are a source of chronic and continual infections (2). They are contributed to a wide variety of bacterial infections, such as gastrointestinal tract, dental plaque, urinary tract infections, and infections of permanent indwelling devices (3). The pathogenicity of *S. aureus* is a complex processor involving a diverse array of virulence factors that are expressed through different stages of infection by a web of virulence regulators (4). The sensing system upregulates different exoproteins related to host-cell damages and downregulates several genes encoding for cell-surface proteins engaged in colonization processes (5). The *agr* system consists of 2 divergently transcribed loci (3 kb) controlled by means of 2 promoters P2 and P3. Most of clinical isolates of acute infections have a functional *agr* system and all, like strains, produce RNAIII *in vitro* and *in vivo* (6). *Agr* deficiency has been related to increased biofilm formation because RNAIII reduces the expression of surface adhesins and increases the production of capsule, toxins, and proteases (7). The *agr* system is supposed to regulate over 70 genes, 23 of which are renowned virulence factors (8). There are 2 classes of virulence factors regulated by *agr*. The first class includes virulence factors implicated in attachment to the host and immune evasion, whereas the second class contains genes engaged in the production of exoproteins related to invasion and toxin production (9). The activation of the *agr* system switches the bacterium from an adhesive, colonizing commensal into an invasive and aggressive pathogen (7). *Agr* dysfunction in the *agr* locus causes changes in the expression of genes and has global effects on bacterial phenotypes including pathogenicity (10). Major virulence factors in *S. aureus*, exfoliative toxins (ETs), toxic shock syndrome toxin (TSST-1), and staphylococcal enterotoxins (SEs) are involved in host colonization, invasion of damaged skin and mucus, gastrointestinal infection, and prevarication of host defense mechanisms (11).

Among metal nanoparticles, AgNPs have a great importance because of their application in various fields as nanomedicines and antimicrobial agents (12). When considering the mechanism for the synthesis of silver nanoparticles, it was hypothesized that the reduction of silver ions was done by enzymes NADH-dependent nitrate reductase (13). Biologically synthesized nanoparticles method is preferred to be compared to chemical and physical approaches because these methods use toxic chemicals, and sometimes reactions occur at a very high temperature (14). However, biological methods for synthesis are eco-friendly, toxic free, and cost-effective in production of AgNPs.

The present study was conducted to estimate the distribution of *agr* among different isolates. The association of operon *agr* with virulence factors (*seb, eta, spa* and *tst v8*) was determined. Moreover, the effect of biosynthesis silver nanoparticles on the function of the *agr* system was studied.

## MATERIALS AND METHODS

### Bacterial isolates.

During December 2015 to April 2016, a total of 100 isolates of *S. aureus* were obtained from 580 patients in different hospitals in Erbil, Iraq. The included specimens were as follow: 120 specimens from urinary tract infections, 65 from nose infections, 127 from wound infections, 78 from burns, 85 from tonsillitis, 45 from vaginitis, and 60 specimens from ear infections. All isolates were identified based on cultural, morphological, and different biochemical tests, in addition to molecular identification using PCR with specific primer to amplify *16SrRNA*.

### DNA isolation.

Bacterial DNA was isolated using Presto Mini™ gDNA bacterial kit (Geneaid, Taiwan).

### Molecular identification.

To confirm the identity of the isolated *S. aureus*, a PCR was applied using specific primer pairs of *16SrRNA* gene, which was designed in this study using primers program on the NBCI website, which was supplied by Bioneer (Korea) as shownin Table 1. This primer amplifies a 1487bp region in *16SrRNA* gene fragment of *S. aureus*. PCR reaction kit was supplied by AccuPower PCR PreMix (Bioneer, Korea). PCR reaction occurred in 20 μL: Primer 2 μL (10 Pmol/μL) of each was forward and reverse, 2 μL of DNA template, and 14 μL of ddH_2_O. PCR amplification was performed using thermal cycler (Eppendorf, Germany) as follows: initial denaturation for 5 min at 94°C, followed by 35 cycles of denaturation at 94°C for 1 min, annealing for 1min at 55°C, and extension for 1:30 min at 72°C, followed by single cycle of final extension for 7 min at 72°C. All PCR products were electro-phoresed in 1.5% agarose gel.

**Table 1.** Primers sequences and their product size

**Primer name**	**Primer sequences**	**Product size (bp)**	**Annealing temperature°C**
*16srRNAF*	CCTGGCTCAGGATGAACG	1478	55
*16srRNAR*	AATCATTTGTCCCACCTTCG		
*agr* F	ATGCACATGGTGCACATGCA	1242	57
*agr* R	CATAATCATGACGGAACTTG		
*seb* F	ACCAGATGAGTTGCACAAAGCGA	342	
*seb* R	TGCTCAGTTACACCACCATACA		
*spa F*	ACCCAAGCCAAAGCGCTAACCT	516	
*spa* R	TGTCAGCAGTAGTGCCGTTTGCT		
*V8* F	TCCACAACAAACGCAGTCAAGCA	654	58
*V8* R	ACACCGCCCCAATGAATTCCGA		
*tst* F	ACACAGATGGCAGCATCAGCCT	128	
*tst* R	AGTTCCTTCGCTAGTATGTTGGCT		
*eta* F	GAGCATGGTCGAAGATGTCGGC	262	
*eta* R	TCCTGCGCCATTCGTTACACTCC		

*16srRNA* gene: 16s ribosomal RNA, R: Reverse; F: Forward, *agr*: accessory gene regulator, *eta*: exfoliative toxin A, *seb*: staphylococcal enterotoxin b, *tst*: toxic shock syndrome toxin, *V8*: serine protease, *spa*: staphylococcal protein

### Quantitative biofilm formation assay.

All isolates were tested for biofilm formation using the quantitative method of Microtitre plate method (MTP) (15), and isolates were classified according to biofilm production.

### Molecular detection of *agr* and virulence factor genes.

The operon *agr* was detected in biofilm-forming *S. aureus* using monoplex PCR as mentioned in PCR amplification for *16SrRNA* gene, with replacing primer for *agr*; and the same PCR program was applied with replacing annealing temperature at 57°C instead of 55°C. Virulence factors genes (*seb, eta, spa, V8, tst*) were detected using multiplex PCR. All designed primers for this study are demonstrated in Table 1.

### Biosynthesis of silver nanoparticles from mushroom.

The fungus used in this study was obtained from local markets in Erbil, Iraq, and was classified as *A. compestris* in Education College/ Salahaddin University, Erbil. The *A. compestris* was washed with distilled water and then dried in shade at room temperature.

Five grams of dried *A. compestris* was powdered into fine particles using an electric grinder and suspended into 350 mL of sterile distilled water and heated for 10 minutes at 55°C. Then, it was filtered using Whatman’s filter paper No. 1 and stored at 4°C.

The prepared extract was used as a reducing agent for 1 mM of AgNO_3_ and for the synthesis of AgNPs as described (16).

### Description of silver nanoparticles.

The preliminary indication for AgNPs production using fungus extract was confirmed by the color change from yellow to dark brown during 1 hour. Moreover, the analysis was performed to identify the absorption of optical spectra of UV-visible spectra, with a resolution of 2.0 nm and between 200 to 600 nm wavelength (16).

Examination of shape and size of the silver nanoparticles manufactured from the reduction of silver nitrate by the fungus extract was done by TEM.

### Determining the minimum inhibitory concentration (MIC) of silver nanoparticles biosynthesis.

The MIC concentration of silver nanoparticles biosyn-thesis was determined using broth dilution method. Different AgNPs concentrations (0.5– 8% v/v) were prepared in 5 mL of Mueller-Hinton broth (Oxoid, England), and the tubes were inoculated with bacterial suspension (about 10^5^ CFU/ml). Then, the tubes were incubated overnight at 37°C, and the MIC was at the lowest concentration of AgNPs, which inhibited visible bacterial growth after overnight incubation.

### The effect of the silver nanoparticles on the function of the *Agr* system.

After determining the MIC of AgNPs, the effect of these particles on the function of the *agr*-quorum sensing system was studied for biofilm forming *S. aureus* (17), with some modification, the inoculate was obtained from the incubated tubes, which contained brain heart infusion broth with sub-MIC concentration of AgNPs plus bacteria. Then, it was cultured on the blood agar plate adjacent to bacterial isolate producing beta-hemolycin at the center of the dish (instead of beta-hemolysin disc) and incubated at 37°C for 24 hours.

### Statistical analysis.

Statistical analysis was performed using chisquare test and the results were considered statistically significant at P< 0.05 level.

## RESULTS

In this study, 100 (17.24%) *S. aureus* strains were isolated from different clinical sources including wound infections (31%), burns (12%), UTI (15%), tonsillitis (8%), vaginitis (3%), ear infections (13%), and nose infections (18%). All isolates of *S. aureus* were identified by conventional phenotypic tests. The results agreed 100% with the results of PCR amplification of 1478 bp fragment of the species-specific gene *16SrRNA* gene.

### Biofilm detection, *agr* gene, and virulence genes.

The results of MtP method revealed that 61% were biofilm producer and 39% were non-biofilm producer isolates. In addition, all biofilm-forming *S. aureus* were tested for *agr* gene using PCR (figure not shown). The results of the present study showed that the number of biofilm-forming *S. aureus* isolates was associated with the type of infection. Chisquare test showed a significant difference at a mean level of less than 0.05 for the distribution percentage of *agr* gene in *S. aureus* isolates, which was isolated from different diseases (Table 2). The percentage of distribution for *agr* gene among different infection cases was 16 (64.00%) for wound infection, followed by urinary tract infection, ear infection, burns, tonsillitis, and nose infection with percentage 5 (55.56%), 2 (50.00%), 4 (44.44%), 2 (40.00%) and 2 (28.57%), respectively. No isolate carried *agr* gene in vaginitis cases (0%). The total number of isolates bearing *agr* was 31 (50.82%).

**Table 2.** Distribution of *agr* gene in biofilm forming *S. aureus* isolates in different sources of infections

**Type of infections**	**No. of biofilm-forming *S. aureus* isolates**	**No. of biofilm-forming *S. aureus* isolates that carried *agr*** gene	**Percentage of biofilm-forming *S. aureus* isolates thatcarried *agr* gene**
Wound infections	25	16	64.00%
Burns	9	4	44.44%
Urinary tract infections	9	5	55.56%
Tonsillitis	5	2	40.00%
Nose infection	7	2	28.57%
Vaginitis	2	0	0.00%
Ear infection	4	2	50.00%
Total number	61	31	50.82%
Chisquare			16.21^*^

* Significance level was set at P< 0.05 level.

The results of some virulence genes (*v8, tst, eta, spa, seb*) in all isolates of biofilm-forming *S. aureus*, using the multiplex PCR, are presented in Table 3.

**Table 3.** The prevalence of virulence genes among *Staphylococcus aureus* isolates

**Type of isolation**	**No. (%) *S. aureus* isolates were carried *tst***	**No. (%) *S. aureus* isolates were carried *eta***	**No. (%)*S. aureus* isolates were carried *seb***	**No. (%)*S. aureus* isolates were carried *spa***	**No. (%)*S. aureus* isolates were carried *v8***	**Chi-square**
*Isolates that carried agr* operon (n=31)	5 (16.13%)	2 (6.45%)	10 (32.26%)	9 (29.03%)	27 (87.10%)	116.09^**^
Isolates that did not carry *agr* operon (n=30)	0 (0%)	0 (0%)	0 (0%)	0 (0%)	0 (0%)	
Total No. 61 (100%)	5 (8.20%)	2 (3.28%)	10 (16.39%)	9 (14.75%)	27 (44.26%)	59.76 ^**^

** Significance level was set at P< 0.05 level.

The results of this study indicated a relationship between *agr*-quorum sensing system and prevalence of virulence genes in isolated *S. aureus*. The prevalence of virulence genes in *agr*- deficient isolates was 0 (0%), while the prevalence of virulence genes in *S. aureus* isolates that carried *agr* gene was 5 (16.13%) for *tst* gene and 2 (6.45) for *eta* gene. Moreover, the prevalence of *v8, spa* and *seb* was 10 (32.26%), 9 (29.03%) and 27 (87.10%), respectively, as presented in Table 3. The results of this study showed that the number of biofilm-forming *S. aureus* isolates was associated with the type of infection. Statistical analysis revealed a significant difference at a mean level of less than 0.05 for the distribution percentage of virulence factor genes in biofilm forming *S. aureus* isolates that carried *agr* operon. Furthermore, a significant difference was found for the distribution percentage of virulence factor gene in all biofilm forming *S. aureus* isolates at a mean level of less than 0.05.

### Silver nanoparticles.

**Fig. 1.**
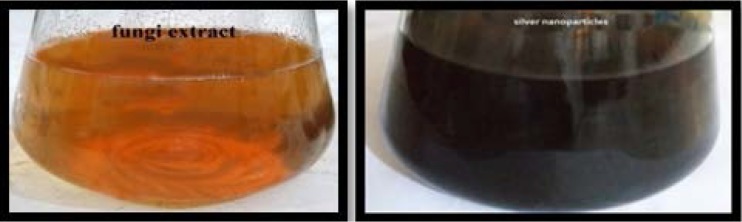
Fungi extract before AgNO_3_ and after addition of AgNO_3_

Silver nanoparticles were visually detected by a change in the color from yellow to dark brown in the reaction suspension, which contained the extract of dried *A. compestris* and silver nitrate (Fig. 1). However, the control did not change its initial color when incubated under the same conditions. The samples containing the synthesized silver nanoparticles showed a peak at 410 nm.

**Fig. 2.**
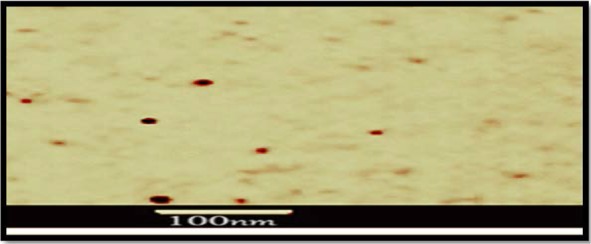
TEM micrograph of AgNPs biosynthesized from Dry *A. compestris*

On the other hand, the characterization of size and shape of silver nanoparticles synthesized by *A. compestris* with transmission electron microscopy (TEM) revealed that the particles were spherical in shape and ranged from 10 to 14 nm (Fig. 2). Also, the MIC of silver nanoparticles was tested against all biofilm forming *S. aureus* isolates that carried *agr* operon, and the results showed that MIC ranged from 8 μg /mL to 64 μg/mL (Table 4).

**Table 4.** Minimum inhibition concentrations of AgNPs nanoparticles against biofilm forming *S. aureus* isolates that carried *agr* operon

**MIC of AgNPs (μg/mL)**	**NO. (%) of isolates**
8	6 (19.35%)
16	13 (41.94%)
32	10 (32.26%)
64	2 (6.45%)

**Fig. 3.**
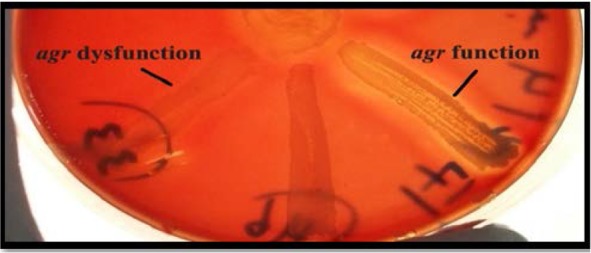
The estimation of operon *agr* efficacy

The results indicated that 31 (100%) isolates had *agr* dysfunction, which was indicated by non-production of delta hemolysin. Also, this study examined the effect of AgNPs on the function of *agr*, and the results showed an activation of *agr* in 31 (100%) biofilm-forming *S. aureus* that carried operon *agr* after treatment with sub-MIC of AgNPs. Then, they were compared with the control treatment, and it was found that there was no change in the function of operon *agr* (Fig. 3).

## DISCUSSION

In the present study, 100 (17.24%) isolates of *S. aureus* were obtained from different clinical specimens by applying phenotypical and biochemical tests, in addition to themolecular identification of PCR and species-specific *16SrRNA* pairs. The MTP method was the most reliable quantitative detection technique *in vitro* that helped the clinicians to make a precise decision about the treatment of such infections. The results of this study showed that 61% of isolates were biofilm producer and 39% were non-biofilm producer isolates. These results were similar with those of a previous study (18) that indicated that 64.89% of *S. aureus* isolates were biofilm producers and 35.11% strains were non-biofilm producers. On the other hand, the results showed that the total number of *agr* gene in *S. aureus* isolates was 31 (50.82%). This result was close to results obtained by Mirani et al. while it was different from the results obtained by Momtaz et al. (19). This difference of obtained results may be due to differences of geographical location and source of isolation. The pathogenicity of *S. aureus* was attributed to the different virulence factors including a large number of cell-surface-bound proteins that were expressed during colonization of the host, and secreted proteins such as hemolysins, proteases, and lipases that were required for acute infections (20). The prevalence of virulence genes in 30 *agr* deficient isolates was 0 (0%). Also, the prevalence of virulence genes in 31 biofilm forming *S. aureus* that carried *agr* gene was 5 (16.13%) for *tst* gene and 2 (6.45) for *eta* gene, respectively, while it was 10 (32.26%), 9 (29.03%), and 27 (87.10%) for *v8, spa* and *seb*, respectively. Abd El-Hamid and Bendary, (21) found that *S. aureus* isolates carried *agr* (100%), *eta* (53.33%), *tst* (33.33%), and *seb* (0%) genes. The results of one study (22) were similar with those of the present study in the proportion of the *V8* gene (51; 92.7%). However, the 2 studies were different in the proportion of *spa, eta* and *tst* genes, which was 55 (100%), 23 (42.0%) and 14 (25.4%), respectively, in the other study. The percentage of *seb* gene (13% vs. 23.6%) was lower in the current study, and the difference in the percentage of virulence genes in the 2 studies may be due to the differences in geographical areas, the source of isolation, and sample size.

Clinicians and researchers have been persuaded to find new alternative molecules to solute the resistant biofilms of pathogenic bacteria (23). Therefore, in this study, the effect of *A. compestris* as AgNPs nanoparticles producer against biofilm forming *S. aureus* isolates was studied. This method was an eco-friendly and cost-effective way of synthesis of silver nanoparticles under laboratory as well as room conditions. The preliminary confirmation of AgNPs from this mushroom species was the color change from yellow to dark brown. The color change was due to the excitation of free electrons present in AgNPs. The intensifies in to brown color after 24 hours (24). The exact mechanism is behind the conversion of AgNo_3_ to silver nanoparticles in mushroom extract are not known, but the extracellular enzymes might be responsible for the biosynthesis of nanoparticles (25). In other studies, the biosynthesis of AgNPs from *Pleurotus florida* and *Microporus xanthopus* was observed at the maximum absorbance peak of 435nm and 425nm, respectively (26).

TEM analysis is a reliable method, which is done on the morphology and size of the nanomaterials. In the present study, the results showed that the size of AgNPs production ranged from 10 nm to 14nm, and it was spherical in shape. The production of AgNPs from *Pleurotus florida* and *Pleurotus sajorcajor* ranged from 20 to 50 nm and 5 to 50nm, respectively (26). The MIC determination test showed the efficiency of these Ag nanoparticles against biofilm forming *S. aureus* isolates that carried operon *agr*. The mechanism of inhibition was unknown, but the possible hypothesis might be due to ionic binding of the AgNPs on the surface of the bacteria, causing an increase in the proton motive force, while the other hypothesis states that the nanoparticles enter into the cell and bind to the enzymes containing thiol groups (27).

Also, the *agr* activity was studied by streaking *S. aureus* isolate adjacent to beta-hemolysin isolate to facilitate the determination of *agr* activity. The results showed that 31 (100%) isolates had *agr* dysfunction, which was indicated by non-production of delta hemolysin. The present study examined the effect of AgNPs on the function of *agr* and the results showed the activation of *agr* in 31 (100%) of biofilm-forming *S. aureus* that carried operon *agr* after treatment with sub-MIC of AgNPs. Then, they were compared with the control treatment and it was found that there was no change in the function of operon *agr.* The phenotypic change in *S. aureus* during infection, from adhesiveness and colonizing to invasiveness and tissue-damaging, is in part coordinated by *agr* operon (1). Therefore, the coordinated regulation of *agr* operon expression is an important criterion for the pathogenic success of *S. aureus* (28). The inactivate *agr* was sometimes found in clinical isolates (2). In addition, *agr* dysfunction has been associated with persistent bacteremia in MRSA.

The final conclusion of this work is that *agr* gene not found in all *S. aureus* clinical isolate and the AgNPs have a good effect on *agr* operon system.
